# Development and storage stability of chickpea, mung bean, and peanut‐based ready‐to‐use therapeutic food to tackle protein‐energy malnutrition

**DOI:** 10.1002/fsn3.2479

**Published:** 2021-07-19

**Authors:** Faiqa Javed, Sidra Jabeen, Mian Kamran Sharif, Imran Pasha, Ayesha Riaz, Muhammad Faisal Manzoor, Amna Sahar, Emad Karrar, Rana Muhammad Aadil

**Affiliations:** ^1^ National Institute of Food Science and Technology University of Agriculture Faisalabad Pakistan; ^2^ Institute of Home Sciences University of Agriculture Faisalabad Pakistan; ^3^ School of Food and Biological Engineering Jiangsu University Zhenjiang China; ^4^ Riphah College of Rehabilitation and Allied Health Sciences Riphah International University Faisalabad Pakistan; ^5^ Department of Food Engineering University of Agriculture Faisalabad Pakistan; ^6^ Faculty of Engineering and Technology Department of Food Engineering and Technology University Gezira Wad Medani Sudan

**Keywords:** chickpea, mung bean, peanut, protein‐energy malnutrition, ready‐to‐use therapeutic food

## Abstract

Protein‐energy malnutrition (PEM) is most prevalent and affecting a large number of children in Pakistan. Ready‐to‐use therapeutic food (RUTF) is a tackling strategy to overcome the PEM in Pakistan. The present research was designed to formulate RUTF from different indigenous sources. After conducting some preliminary trials, 14 RUTF formulations were developed by mixing peanut, mung bean, and chickpea alone as well as in various combinations with the addition of sugar, powdered milk, oil, and vitamin‐mineral premix. Freshly prepared RUTF was stored at room temperature (20 ± 5°C) and packed in aluminum foil for 90 days to investigate the microbiological analysis (total plate count and mold count), water activity (A_w_), peroxide value, and thiobarbituric acid (TBA) value. All the parameters showed significant (*p* < .05) differences among peanut, chickpea, and mung bean‐based RUTF except water activity. The storage days and interaction between treatments and storage days also showed a significant (*p* < .05) effect on water activity, total plate count, mold count, peroxide value, and TBA of RUTF formulations. The present study revealed that the peanut, chickpea, and mung bean can be used in the formulation of RUTF due to their shelf stability and help to mitigate the PEM in Pakistan.

## Graphical abstract

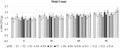

## 1 INTRODUCTION

Malnutrition is a widely prevalent nutritional dilemma in developing countries. This mostly happens due to nonoptimal consumption and utilization of nutrients essential for various physiological processes (Endris et al., 2017). Nearly half a billion children with age below 5 years are undernourished and most of them belong to South Asia and Africa. The United Nations International Children's Fund (UNICEF) has reported 1 out of 13 children being challenged by wasting. Pakistan, India, and Bangladesh are the home of almost half of the global malnourished individuals (Asim and Nawaz, 2018). The ever‐increasing population and high cost of animal‐derived foods have made it necessary to demand the development of cheap plant‐based nutritious foods to counter this problem (Awan, 2011; Naeem et al., 2021; Shahzad et al., 2021; Waseem et al., 2021).

Protein‐energy malnutrition (PEM) is a broad term and comprehends various pathological conditions arising due to concurrent lack of energy‐ and protein‐dense diets. This deficiency makes malnourished infants as well as young children more prone to the onset of diseases and infections. Consequently, these infectious diseases are responsible for about two‐thirds of all the mortalities in children under 5 years of age residing in developing homelands (Demissie & Worku, 2013). There is also altered metabolism of proteins and essential amino acids characterized by decreased levels of circulatory plasma proteins and albumin (Molfino et al., 2014). The consequences of malnutrition in children include the presence of pneumonia, diarrhea, fatigue, delayed growth, metabolic disorders, cognitive development, reduced economic productivity, and even mortality (Roger et al., 2015). The primary cause of malnutrition in Pakistani children is the poor quality of their diets and less availability of supplementary foods.

The national and global nutrition surveys have documented an array of nutritional discrepancies among the various segments of the population in Pakistan mainly attributed to insufficient intake of quality protein and balanced diets. The consumption of legumes, as well as their incorporation in dietary staples, may help alleviate protein‐energy malnutrition (Papalamprou et al., 2010). FAO (2010) has greatly stressed exploiting indigenous sources of plant protein to enhance the availability of cost‐effective food products. The protein obtained from animal sources is expensive and therefore is inaccessible to the masses in developing countries. These plant sources can be combined in different combinations with another foodstuff to make them nutritious and balanced part of a meal (Day, 2013). Legumes are the potential ingredient to get the protein‐enriched inexpensive and sustainable infant diets (Maphosa & Jideani, 2017).

It is the need of the hour to manufacture infant formulas, complementary foods, and supplements for vulnerable segments of the population using locally available high‐quality raw materials as a substitute to expensive animal‐based proteins and imported stuff. In this context, legumes are promising ingredients. These are seeds or fruits belonging to the family Fabaceae (*Leguminosae*) and are grown primarily for their seeds known as pulse, crop for livestock silage/forage, and soil‐enhancing green manure. These include chickpea, mung bean, mash, soybean, peanut, peas, and lentils. Traditionally, pulses are considered an important part of human nutrition (Ijarotimi & Keshinro, 2012). While in Pakistan, pulses consumption is extremely low, *that is*, 6–7 Kg per person‐year. Additionally, legumes have a lower ecological impact than other protein‐enriched sources (Asif et al., 2013).

The consumption of legumes could be helpful in the reduction of malnutrition in poor masses (Maphosa & Jideani, 2017). Chickpea (*Cicerarietinum L*.*)* is one of the most utilized ingredients in the world. It is an essential component of stews, soups, and salads in African countries, whereas in Asia, it is used in roasted, salted, and boiled forms (Hirdyani, 2014). Mung bean (*Vigna radiata)*, a tropical legume is utilized in various cookeries, *for example*, porridge, pastry, and sauces globally (Hussain & Burhanddin, 2011). Unfortunately, no infant formula is being manufactured in the country from indigenous resources. Hence, only people with strong financial resources can buy imported formulas (Dube et al., 2009).

Ready‐to‐use therapeutic food (RUTF), a usual pack, includes skim milk powder, whey powder, and peanut as protein sources, sugar (carbohydrate source), edible oil (fat source), and desired levels of minerals and vitamins. According to WHO, it must provide about 41%–58% carbohydrates, 13%–16.5% protein, 26%–36.7% lipids, and <5% fiber. It is regarded as a shelf‐stable product due to low moisture content (maximum moisture 2.5%) and water activity (<0.6). The lives of thousands of severely malnourished children per year have been saved after the development of peanut‐based RUTF. The major benefit of RUTF is its ready‐to‐use nature as nothing is required for its preparation before serving. It does not require refrigeration, hence equally suitable for tropical environments. Its shelf life stability could be further enhanced due to better packaging and storage conditions (Wakhu‐Wamunga & Wamunga, 2017). The present study has been conducted to develop different proto‐types of RUTF from indigenous sources (chickpea and mung bean) for the treatment of malnourished children. Furthermore, the developed formulation was optimized and evaluated for storage and shelf life stability.

## 2 MATERIALS AND METHODS

Mung bean (*Vigna radiatus)* chickpea (*Cicer* *arietinum*) and peanut (*Pyrus communis*) were procured from Ayub Agriculture Research Institute, Faisalabad, Pakistan. Sugar, vegetable oil, and milk powder were procured from Metro‐Cash and Carry, Faisalabad, Pakistan. The vitamin/mineral premix purchased from Fortitech Inc. All reagents of analytical grade purchased from Merck and Sigma‐Aldrich and Fisher Scientific (CHEMTREC^®^). Peanuts were de‐hulled manually to obtain peanut kernels, and all ingredients (mung bean, chickpea, and peanut kernels) were homogeneously mixed according to the formulation of RUTF (Table 1) and converted into uniform powder using Udy Cyclone Mill and sieved. All samples were packed in airtight plastic bags, sealed, and stored at ambient temperature (20 ± 5°C) for 90 days.

**TABLE 1:** Treatments used in study

Treatments^a^	Peanut (%)	Chickpea (%)	Mung bean (%)
T_0_	100	–	–
T_1_	80	20	–
T_2_	60	40	
T_3_	40	60	–
T_4_	20	80	–
T_5_	–	100	–
T_6_	80	–	20
T_7_	60	–	40
T_8_	40	–	60
T_9_	20		80
T_10_	–	–	100
T_11_	–	20	80
T_12_	–	40	60
T_13_		60	40
T_14_	–	80	20

Note

T_0_ = RUTF with 100% peanut act as control; T_1_ = RUTF with 80% peanut and 20% chickpea; T_2_ = RUTF with 60% peanut and 40% chickpea; T_3_ = RUTF with 40% peanut and 60% chickpea; T_4_ = RUTF with 20% peanut and 80% chickpea; T_5_ = RUTF with 100% chickpea; T_6_ = RUTF with 80% peanut and 20% mung bean; T_7_ = RUTF with 60% peanut and 40% mung bean; T_8_ = RUTF with 40% peanut and 60% mung bean; T_9_ = RUTF with 20% peanut and 80% mung bean; T_10_ = RUTF with 100% mung bean; T_11_ = RUTF with 20% chickpea and 80% mung bean; T_12_ = RUTF with 40% chickpea and 60% mung bean; T_13_ = RUTF with 60% chickpea and 40% mung bean; T_14_ = RUTF with 80% chickpea and 20% mung bean.

^a^ Micronutrients were added in all formulations according to standard for RUTF.

### 2.1 Preliminary steps in the development of RUTF

A laboratory‐scale mixer was used for homogenous mixing of all the dry ingredients. Oil was added directly into the mixing bowl followed by mixing of the raw ingredients at different levels of peanuts, mung beans, and chickpeas to form a uniform paste of thick consistency (Table 1). A premix of vitamins/minerals was added during the blending process in all newly developed formulations. These products were packed in heavy‐duty aluminum foil, sealed, and stored at ambient temperature (20 ± 5°C) for 90 days and used for further study.

### 2.2 Development of RUTF

The RUTF was developed following the modified methods of Manary et al. (2004) and Ciliberto et al. (2005). All powdered ingredients (peanuts, mung beans, and chickpeas) were blended at different proportions (Table 1) and shifted into Planetary Bakery Mixer (A‐200). During this process, weighed amounts of powdered milk and sugar were also added as per RUTF specifications (Table 2) followed by the addition of vitamins and mineral premix while edible oil was added at the end of the blending process (vigorous stirring for 6–7 min). A semi‐soft, homogenized paste having thick consistency was obtained. After confirmation that homogenized paste has formed properly and will not be separated during storage, RUTF sachets (Weighing ~100 g) were sealed using Vacuum Sealer (PFS‐200, Impulse Sealer), labeled, and stored at ambient temperature (20 ± 5°C) for 90 days in cardboard boxes. These samples were tested for microbiological analysis, water activity, peroxide value, thiobarbituric acid (TBA) value as well as color analysis following their respective methods.

**TABLE 2:** RUTF formulation used in study

Ingredients	Weight (g)
Defatted peanuts/chickpeas/mung bean	50 (as per treatment plan)
Sugar	13.4
Oil	25
Powdered milk	10
Vitamin and mineral premix	1.6

### 2.3 Microbiological analysis

All samples of RUTF were analyzed for total plate count and mold count according to Method no. 42–15.02 and 42–50.02, respectively, as given in the (AACC 2000). Arithmetic mean was calculated as a total number of bacteria cfu/g of sample and total mold count per plate.

### 2.4 Water activity

The water activity (A_w_) of all RUTF samples was determined using a water activity meter (Rotronic HygroPalm HP23‐AW‐A‐set‐14) following the procedure of El‐Nimr et al. (2010). The meter was first standardized using 6 mol/kg sodium chloride and 13.41 mol/kg lithium chloride.

### 2.5 Thiobarbituric acid

Thiobarbituric acid (TBA) was measured as stated by Kirk and Sawyer (1991) to evaluate the shelf stability of the developed products. RUTF sample (10 g) was taken in the distillation flask and heated to obtain 50 ml distillate in glass stoppered test tube with 50 ml TBA reagent (0.2883 g/100 ml of 90% glacial acetic acid) followed by the heating in the water bath for 35 min. A blank sample was used to measure the absorbance (D) through a spectrophotometer (VIS‐1100, Biotechnology Medical Services) at 538 nm. The TBA was calculated according to the following formula:

$$\text{TBA}\;\text{no}\frac{(\text{Malonaldehyde}\;(\text{mg}))=7.8\times }{\text{RUTF}\;(g)}$$

### 2.6 Peroxide value

Ready‐to‐use therapeutic food was studied for peroxide value according to Method no. 920.160 as given in AOAC (2016). RUTF (5 g) was taken into Erlenmeyer flask (250 ml) containing glacial acetic acid‐chloroform (30 ml of 3:2v/v) solution and shake for 1 min. Freshly prepared saturated solution (0.5 ml) of potassium iodide was mixed and titrate against 0.1 N solution of sodium thiosulphate (Na_2_S_2_O_3_) till the yellowish color was disappeared. Thereafter, starch solution (0.5 ml) was added and titration was continued till the yellow color was disappeared. Iodine produced during reaction was used as an index of peroxide value. Blank reading was noted and peroxide value calculated by using the following formula:

$$\text{Peroxide}\;\text{value}=\frac{(B‐A)\times N\;}{\text{RUTF}\;(5g)}\times 1000$$

B = Volume of Na_2_S_2_O_3_ used for blank.

A = Volume of Na_2_S_2_O_3_ used for sample.

N = Normality of Na_2_S_2_O_3_.

### 2.7 Statistical analysis

Data were statistically interpreted by two‐way analysis of variance using Statistix 8.1 version computer software (Statistix 8.1). The difference in all RUTF samples was compared at the 5% level of significance using a completely randomized design (CRD) design.

## 3 RESULTS AND DISCUSSION

### 3.1 Water activity (A_w_)

**FIGURE 1:**
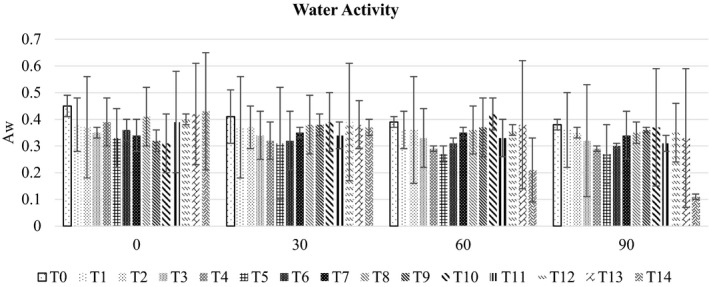
The bar graph showing the effect of storage on water activity of different RUTF formulations

The results regarding the water activity (Figure 1) showed a significant difference (*p* < .05) among all RUTF formulations (0.28 ± 0.15–0.39 ± 0.02) with different blends of peanut, chickpea, and mung bean. The freshly prepared RUTF exhibited the highest water activity A_w_ (0.39 ± 0.03 overall means for all treatments). After 30 days of interval, A_w_ was gradually decreased to 0.36 ± 0.03, 0.34 ± 0.05, and 0.32 ± 0.07 respectively, during the storage period of 90 days. It is obvious from the results that RUTF was shelf‐stable as the existing A_w_ value was not supportive of the growth of any type of microorganisms.

The shelf life of RUTF usually ranged from 12–24 months (Hossain et al., 2020). Their prolonged shelf life is mainly due to a nonsupportive environment for bacterial growth (Latham et al., 2011). Additionally, contamination during storage is hindered by the proper packaging. In a study, rice, barley, and maize‐based RUTF was assessed for water activity. The results showed 0.290, 0.279, and 0.260 water activity in rice‐sesame, barley‐sesame, and maize‐sesame‐based RUTF, respectively, as compared to 0.241 in Plumpy'nut (Collins, 2007). In this study, groundnut, chickpea, and mung bean were the major raw ingredients used in RUTF formulations. Overall, chickpea and mung beans have excellent water absorption properties making the moisture content unavailable for microorganisms. This is also evident from the increase in the hardness of RUTF samples during the storage which was more in chickpea and mung bean‐based formulations than that of RUTF containing 100% peanut. Mohammed et al. (2014) study the impact of chickpea flour on pasting and bread‐making quality. The addition of chickpea flour improved the water absorption of the respective bread samples.

### 3.2 Total plate count

**FIGURE 2:**
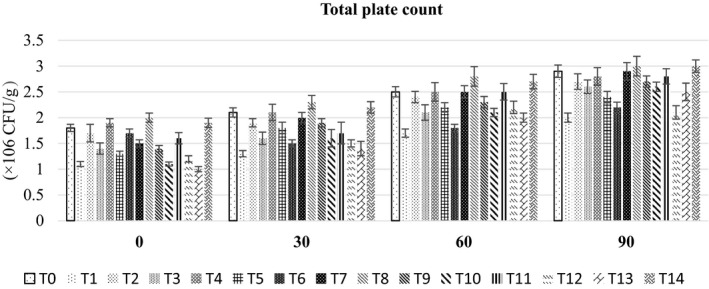
The bar graph showing the effect of storage on total plate count of different RUTF formulations

Means for TPC (Figure 2) showed a higher count (2.53 × 10^6^ ± 0.12 CFU/g) in the RUTF developed with 40% peanut and 60% mung bean (T_8_). Overall, RUTF developed with peanut and mung bean showed TPC ranging from 2.53 × 10^6^ ± 0.12 CFU/g to 2.30 × 10^6^ ± 0.10 CFU/g. Chickpea‐ and mung‐based RUTF exhibited TPC ranged from 1.73 × 10^6^ ± 0.08 CFU/g to 2.45 × 10^6^ ± 0.12 CFU/g. Among the RUTF developed with peanut and chickpea, the maximum total plate count (2.30 × 10^6^ ± 0.10 CFU/g) was noticed in RUTF developed with 20% peanut and 80% chickpea, whereas minimum value (1.53 × 10^6^ ± 0.07 CFU/g) was in RUTF having 80% peanut and 20% chickpea. RUTF developed with peanut, chickpea, and mung bean showed a minimum value of total plate count (1.51 × 10^6^ ± 0.32 CFU/g) at 0 days followed by 1.79 × 10^6^ ± 0.31 CFU/g at 30 days, 2.29 × 10^6^ ± 0.31 CFU/g at 60 days and 2.67 × 10^6^ ± 0.28 CFU/g) at 90 days of storage. The findings of the present research are in line with the earlier studies carried out on RUTF. In research, cereal and legume‐based ready‐to‐use supplementary food (RUSF) was developed for malnourished children. The total plate count of these formulations was 1,850 CFU/g which was within the acceptable limit specified by the WHO, WFP, and UNICEF, *that is*, 10^4^ Cfu/g (UNICEF, 2007). The mung bean and soy‐based RUTF developed for HIV‐positive patients in Vietnam showed a 450 CFU/g micro‐organism count which is far below that of UNICEF specifications of RUTF, that is, <10,000 CFU/g (Wieringa et al., 2013).

### 3.3 Mold count

**FIGURE 3:**
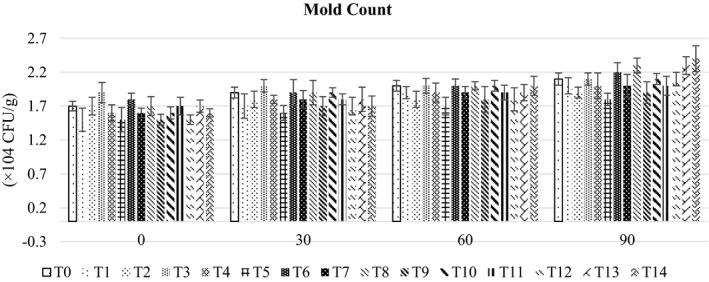
The bar graph showing the effect of storage on mold count of different RUTF formulations

Mean mold count (Figure 3) showed the highest value (2.00 × 10^4^ ± 0.09 CFU/g) in RUTF made with 40% peanut and 60% chickpea, whereas the lowest value (1.65 × 10^4^ ± 0.07 CFU/g) was observed in RUTF developed with 100% chickpea. Among the chickpea and mung beans‐based RUTF, maximum value (1.93 × 10^4^ ± 0.07 CFU/g) was observed in 60% chickpea and 40% mung bean, whereas minimum value (1.78 × 10^4^ ± 0.10 CFU/g) was noted in 40% chickpea and 60% mung bean. Similarly, peanut‐ and chickpea‐based RUTF showed minimum (1.78 × 10^4^ ± 0.11 CFU/g) mold count in 80% peanut and 20% chickpea, whereas maximum value (2.00 × 10^4^ ± 0.09 CFU/g) was observed in 40% peanut and 60% chickpea. Likewise, peanut and mung bean‐based RUTF showed maximum (1.98 × 10^4^ ± 0.10 CFU/g) mold count in 80% peanut and 20% mung bean whereas minimum value (1.73 × 10^4^ ± 0.07 CFU/g) was observed in 20% peanut and 80% mung bean.

Yeasts and molds are ubiquitous in the environment and can contaminate food through inadequately sanitized equipment or as airborne contaminants. Yeast and molds count frequently predominate when conditions for bacterial growth are less favorable, such as lower water activity, low pH, high salt, or high sugar content (OECD, 2011). The yeast and mold count in legume, cereal, and seed‐based RUSF were found to be 48 CFU/g which was within the acceptable limit of 50 CFU/g in the product (UNICEF, 2007). Storage exhibited a significant impact on mold count. In fresh samples, it was (1.64 × 10^4^ ± 0.12 CFU/g at 0 days, 1.80 × 10^4^ ± 0.11 CFU/g) at the 30th day, 1.91 × 10^4^ ± 0.10 CFU/g at 60th day, and 2.08 × 10^4^ ± 0.17 CFU/g at 90th day of storage. During storage, the increase in mold growth for different treatments is in line with the results of earlier studies. In a study, date‐based products like chutney and date relish were assessed for microbial growth. The mold count of developed products was increased from 2.40 to 5.83 CFU/ml during 5 months of storage (Al‐Hooti et al., 1997). In a study, cereal and legume‐based RUSF were evaluated for their microbiological quality. The results showed a 48 CFU/g mold count in developed products (Niraula, 2018). Likewise, Bako (2018) has reported a maximum of 50 molds per g in RUTF.

### 3.4 Thiobarbituric acid number

**FIGURE 4:**
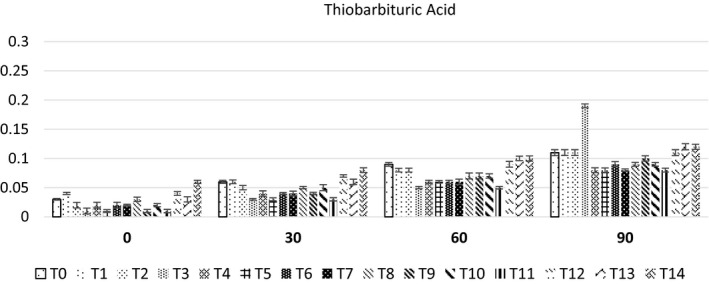
The bar graph showing the effect of storage on TBA of different RUTF formulations

Means for thiobarbituric acid number (Figure 4) showed the maximum value in T_14_ (0.06 ± 0.005) mg malonaldehyde/Kg of the sample) RUTF with 80% chickpea and 20% mung bean, whereas it was lowest (0.01 ± 0.005 mg/Kg) in RUTF prepared with 20% chickpea and 80% mung bean (T_11_). Similarly, peanut and chickpea‐based RUTF showed minimum (0.02 ± 0.009 mg/Kg) thiobarbituric acid number in 20% peanut and 80% chickpea while the highest value (0.04 ± 0.003 mg/Kg) was found in 80% peanut and 20% chickpea. Likewise, peanut and mung bean‐based RUTF showed maximum (0.03 ± 0.007 mg/Kg) TBA in 40% peanut and 60% mung bean, whereas minimum value (0.01 ± 0.005) was observed in 20% peanut and 80% mung bean. Storage study also exhibited a significant impact on the thiobarbituric acid number of RUTF. At the initiation of storage, TBA value was (0.02 ± 0.006 mg/kg) which was gradually increased to 0.05 ± 0.005, 0.07 ± 0.006, and 0.10 ± 0.007 mg/Kg) for 90 days storage. TBA number is considered as a standard marker for the determination of lipid peroxidation changes during storage (MANZOOR et al., 2021). According to Kirk and Sawyer (1991), the refined oil has 0.02–0.08 TBA value in a good condition while it exhibits a value of 0.1–0.2 mg malonaldehyde per Kg in badly stored oil. The porridge developed from groundnut, and maize composite flours showed 0.27–1.92 TBA, while during 90 days of storage, it was elevated from 0.28 to 4.53 mg malonaldehyde per Kg (Temba et al., 2016).

### 3.5 Peroxide value

**FIGURE 5:**
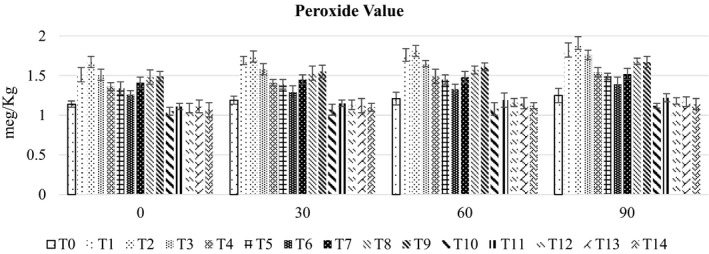
The bar graph showing the effect of storage on peroxide value of different RUTF formulations

Means for the peroxide value (Figure 5) revealed the highest amount (1.78 ± 0.07 meg/Kg) in RUTF prepared with 60% peanut and 40% chickpea followed by 1.08 ± 0.05 meg/Kg in RUTF containing 100% mung bean while the lowest value 1.11 ± 0.09 meg/Kg was in RUTF developed with 80% chickpea and 20% mung bean. Among the chickpea and mung beans‐based, RUTF maximum peroxide value (1.17 meg/Kg ± 0.05) was observed in 20% chickpea and 80% mung bean, whereas minimum value (1.11 ± 0.09 meg/Kg) was noted in 80% chickpea and 20% mung bean. Similarly, peanut and chickpea‐based RUTF showed minimum (1.45 ± 0.04 meg/Kg) peroxide value in 20% peanut and 80% chickpea, whereas maximum value (1.78 ± 0.07 meg/Kg) was observed in 60% peanut and 40% chickpea. Likewise, peanut and mung bean‐based RUTF showed maximum (1.58 ± 0.09 meg/Kg) value in 20% peanut and 80% mung bean, whereas minimum value (1.32 ± 0.09 meg/Kg) was observed in 80% peanut and 20% mung bean. Storage also has a significant effect on the thiobarbituric acid number of RUTF.

Peroxide value is the concentration of peroxides and hydroperoxides developed in the starting phase of lipid oxidation employed for assessing oxidative rancidity. Milliequivalents of peroxide per Kg of fat are measured by titration with iodide ion. Higher values of peroxides are an indicator of fat rancidity, as well as moderate values produce peroxides depletion after reaching high concentrations (Pizarro et al., 2013). Fats and oils play a vital part in taste, texture, texture, and improving the nutritional quality of different foods (Asif, 2015). The acceptable limit of POV fixed by the European Union (EU) for the freshness of food is 10 meq/Kg. The porridge prepared by use of maize and groundnut composite flours showed 0–0.31 meq/Kg POV in maize flour and 0.02–0.88 meq/Kg in full‐fat groundnut for 90 days storage (Temba et al., 2016).

## 4 CONCLUSION

All newly formulated RUTF (T_0_‐T_14_) showed a good shelf life acceptability and water activity of less than 0.5. The combination of chickpea and mung bean improved the protein content that helps to increase the nutritional quality and textural properties of RUTF. The microbial count and water activity (A_w_) decreased during storage has presented the good shelf stability of the product. Based on storage stability and better retention of nutritional quality, the three best RUTF formulations, *that is*, G_1_ (RUTF with 100% chickpea), G_2_ (RUTF with 80% chickpea and 20% mung bean), and G_3_ (RUTF with 40% chickpea and 60% mung bean), were selected. These findings demonstrated that the newly formed RUTF can be used as a replacement for acute, moderate, and severely malnourished infants of age ranging 0–59 months. The most common health problem faced in Pakistan is EM. Therefore, these plant‐based RUTF can be used to combat the situation of malnutrition.

## AUTHOR CONTRIBUTIONS

**Faiqa Javed:** Conceptualization (equal); Formal analysis (equal); Methodology (equal). **Sidra Jabeen:** Formal analysis (equal); Investigation (equal); Methodology (equal); Writing‐original draft (equal). **Mian Kamran Sharif:** Supervision (equal); Writing‐review & editing (equal). **Ayesha Riaz:** Data curation (equal); Software (equal). **Muhammad Faisal Manzoor:** Data curation (equal); Software (equal); Writing‐review & editing (equal). **Emad Karrar:** Data curation (equal); Software (equal). **Rana Muhammad Aadil:** Software (equal); Supervision (equal); Writing‐review & editing (equal).
